# On the issue of costs in programmatic assessment

**DOI:** 10.1007/s40037-016-0295-z

**Published:** 2016-09-15

**Authors:** Cees P. M. van der Vleuten, Sylvia Heeneman

**Affiliations:** 1 Department of Educational Development and Research, Faculty of Health, Medicine and Life Sciences, Maastricht University, Maastricht, The Netherlands; 2 Department of Pathology, Faculty of Health, Medicine and Life Sciences, Maastricht University, Maastricht, The Netherlands

**Keywords:** Programmatic assessment, Cost, Resources

## Abstract

Programmatic assessment requires labour and cost intensive activities such as feedback in a quantitative and qualitative form, a system of learner support in guiding feedback uptake and self-directed learning, and a decision-making arrangement that includes committees of experts making a holistic professional judgment while using due process measures to achieve trustworthy decisions. This can only be afforded if we redistribute the resources of assessment in a curriculum. Several strategies are suggested. One is to introduce progress testing as a replacement for costly cognitive assessment formats in modules. In addition, all assessments should be replaced by assessment formats that are maximally aligned with the learning tasks. For performance-based assessment, OSCEs should be sparsely used, while education and work-embedded assessment should be maximized as part of the routine of ongoing instruction and assessment. Information technology may support affordable feedback strategies, as well as the creation of a paper trail on performance. By making more dramatic choices in the way we allocate resources to assessment, the cost-intensive activities of programmatic assessment may be realized.

## Programmatic assessment

Programmatic assessment is a holistic approach to assessment [1–3]. As in a curriculum design, the approach to assessment is a planned one with deliberate choices of assessment methods, assessment scheduling and assessment feedback strategies. Any method of assessment may be used in programmatic assessment. The choice of method depends on the educational justification for using that method at that moment of time. Each individual assessment is seen as a single data point. Each individual data point is optimized for learning, meaning that the assessment task should be strongly aligned to the education task and that the feedback is meaningful to the learner. The summative/formative distinction is reframed as a continuum of stakes, ranging from low-stakes to high-stakes assessment. Decision-making on learner progression is proportionally related to the stakes. Higher-stakes decisions can only be taken with sufficient data points. Each data point is low stakes because pass/fail decisions are removed from it. The focus is exclusively on information provision. Years of research in education and other fields has revealed the critical importance of feedback for learning [4]. Learners are supported in their use of feedback and their self-directed learning through mentors who build an entrusted relationship with the learner. High-stake decisions, i. e. promotion to the next year or graduation, are taken by an assessment committee with sufficient independence from the mentor and the learner. Procedural and process measures are taken in such a way that these high-stake decisions are trustworthy, for example by triangulation of information, member checking, or creating an audit trail [5]. Programmatic assessment is based on an interpretation of decades of assessment research [6].

## Programmatic assessment is costly

Whenever we present programmatic assessment, one of the first questions that invariantly emerges is cost. With the limited time and resources available in education, whether undergraduate or postgraduate, how can we find the means for the provision of feedback, the mentoring of students and solid professional judgment decisions on progress? Indeed, whether programmatic assessment will work or not strongly depends on the richness of the feedback the assessment generates. Feedback may be quantitative or qualitative. Quantitative feedback can be provided digitally and inexpensive online systems may be developed [7]. Detailed scoring reports may be provided on relevant topics addressed in the assessment, benchmarked to a reference group. The more complex the skill, however, the less informative scores become. Complex skills such as academic writing, communication, collaboration, professionalism, etcetera benefit more from feedback in the form of words or narratives than from scores [8, 9]. If feedback is not credible, learners will ignore it [10]. Getting teachers or clinical supervisors to provide credible narratives is a challenge and requires time and effort. So does mentoring. Mentoring has shown to be a very effective instructional strategy and promotes self-direction of learning [11]. Much of the feedback given to learners is ignored [4, 12] and creating follow-up on feedback, in the form of a dialogue, is helpful for feedback uptake [13]. Another costly element in programmatic assessment is the committee that makes high-stake decisions. Human professional judgement is inevitable when quantitative and qualitative information needs to be aggregated. However, the committee work can be organized very efficiently. Deliberation is limited to only those learners where there is doubt on the clarity of the information in relation to a pass/fail decision. Nevertheless, all these elements in programmatic assessment are intense and require time and effort, a commodity that is sparse in education. So how can we afford programmatic assessment in our normal education practice?

## Making thoughtful choices

Our central argument is a careful redistribution of resources in assessment. Data on cost estimates of assessment are rare, however. So far, no one has published about costs in programmatic assessment. We would argue that the expenditure is mostly in people costs (feedback, mentoring, judging about progress). Clearly good assessment is costly. Actually, assessment is only as good as the effort and resources one is prepared to put into it [6, 14]. We argue that we waste a lot of resources in assessment [15] and that we can take sharp cost reduction measures. The resulting savings can be reinvested in the costly components of programmatic assessment.

We are strong proponents of progress testing [16]. With progress testing one periodically assesses all the students in the curriculum and one monitors the growth in applied knowledge on all content areas of a curriculum. It also has many educational advantages, such as a rich source of feedback, deeper learning, and curriculum benchmarking. Progress testing is perceived to be costly, but it is not. Table 1 provides a rough estimate of progress tests and module tests using our own university staff expenditures, assuming the use of multiple-choice questions in both formats. Module test costs are three times higher than the cost of progress tests. This is due to the student multiplier. In progress tests all students participate in a single test whereas only one cohort is tested by a module test. Other running costs such as test administrative, infrastructural and resit examination costs are excluded from the calculation. These costs would even widen the gap in favour of progress testing. Standard setting costs are excluded as well. Costly standard setting methods (i. e. Angoff procedures) would widen the gap even further. We acknowledge that our comparison is not based on a thorough analysis of actual costs. We call for more studies on cost, not so much of individual tests but on assessment programmes as a whole. This would require a more in-depth knowledge of the time and resources involved in running an assessment programme. Perhaps we can also borrow some theory and tools from economists on cost-benefit analyses [17] or from economic studies on healthcare provision [18].

**Table 1:** Estimation of staff costs per student per test for progress tests and module tests consisting of multiple-choice questions in a six-year undergraduate curriculum, excluding test administration, infrastructural and standard setting costs

Source of cost	Calculation	fte	€
*Progress test*	200 items × 4 tests per year × 1 × k€ 100 h	0.48	48,485
Item production	Chair and 5 members	1.00	100,000
Admin support	k€ 60 h	1.00	60,000
Total	–	2.48	208,485
*Module test*			
Item production	6 tests × 60 items each × 1 h × k€ 100 h	0.22	21,818
Review committee	Dispersed committees across tests	0.5	50,000
Admin support	k€ 60 h	1.5	90,000
Total	–	2.22	161,818
–		Cost per test per learner	
		Progress	Module
Cohort size per year		Test	Test
100		€ 87	€ 270
200		€ 43	€ 135
300		€ 29	€ 90
400		€ 22	€ 87

The estimate is rough indeed. It assumed that new tests are being made every year. This may not be the case for module testing in many practices. On the other hand, resit examinations are excluded for module tests (they do not exist for progress tests). Running costs are also excluded (test administration and infrastructural costs). Our own progress test was developed in collaboration with five other medical schools, so the cost is even further reduced. The cost calculation is only exemplary to point to a cost difference between the two approaches of assessment, not to claim much accuracy

Apart from the comparative cost, the more interesting question though is what progress testing may replace? Progress testing provides a pretty robust picture on how a learner develops in the cognitive domain. There is not much point in having module-related assessments repeating what is assessed in a progress test. For example, in one school of medicine, progress tests are the only form of cognitive testing, with the exception of one single first year test in the cognitive domain [19]. The amount of resources being saved in this strategy is just phenomenal, as can be seen from Table 1. A very smart and radical choice is to free up time and resources for other assessment activities! In recent research it was found that learners want two most dominant elements to be realized in an assessment programme: agency (the extent to which the assessment reflected personal learning) and authenticity (assessment representing relevant tasks for becoming a good doctor) [20]. To reward individual learning activities and using the testing effect [21], we would recommend module-based assessment activities that are closely linked to the individual learning tasks in a module, and are authentic to what skills need to be addressed in the module, but that do not mimic what is already being tested in a progress test. Examples are time-dispersed mini-tests [22], assessments of learning products or oral examinations evaluating individual learning experiences, and peer and tutor assessments. When this is done as part of the ongoing learning and as low-stake data points, this will contribute to reinforcing the intended learning tasks and their resulting learning behaviour [23]. If you insist on using a standard setting for defining substandard performance, use cheap methods of standard setting such as, for example, the Cohen method [24].

Another strategy of reducing cost would be to share our standardized testing material across the world. We are all engaged in the same assessment activities and we all reinvent the same wheel at a substantial price. Facilitating and sharing of test material would probably have a substantial impact on reducing the cost of standardized assessment. It is time to explore this and use our strong networks in the medical education community.

When we wish to assess behavioural competencies, either in school or in the workplace, observation is the preferred method and the OSCE is a commonly used method. The OSCE is also very expensive [25]. We would encourage to be sparse with OSCEs. In our view OSCEs are relevant when learning is still done in a simulated environment such as in a simulation or skills centre. As soon as learning takes place in a real, workplace-based environment, we would encourage evaluating the habitual performance in that environment (please note that many behavioural skills can also be demonstrated outside a clinical environment within a school, e. g. professional behaviour assessment in a tutorial PBL group). Over recent years we have acquired a formidable amount of knowledge on how to assess habitual performance [6]. Providing holistic professional judgement and capturing feedback on observed activities is the hallmark of these assessment activities. The value of these assessments strongly depends on the quality of the interaction between the feedback giver and the feedback receiver and the way this information is logged into a paper or computer trail. Indeed, this assessment format is costly. However this assessment format is fully part of the ongoing instruction process and addresses the often expressed need of learners for feedback but not getting it, particularly in workplaces [26, 27]. To get this assessment format right and affordable, it should be embedded within practice routine and use as little teacher time as possible. By making assessment part of a routine it will become a normal activity, not something that is an add-on and estranged to core professional activities. If done properly, learners will become engaged and will want more feedback [28]. Learners may be empowered to ask and log the feedback. Technology may be used to capture feedback in a time-efficient way, such as reflection apps and handheld IT devices [29]. In summary, when assessing performance we would encourage reducing costly simulated standardized assessment to a minimum and only where it has added value. All other performance assessment should be education or work embedded as part of the natural and routinely occurring learning processes. The paper trail that is created from this type of assessment will provide a solid basis for making inferences on complex behavioural skills of learners much better than any standardized single moment of assessment is able to do.

## Conclusion

Programmatic assessment is affordable if we make sharp decisions on where to spend our resources on assessment. We have given some thoughts on how to make such decisions. In educational practice we are often captured in ritualistic and unrevealed expensive assessment strategies where every part of the curriculum is assessed in the same summative way. By thinking out of the box, by looking at the assessment as an integral and holistic approach, we should be able to make smarter and more inexpensive decisions. We should intensify where it matters most – personalized feedback, guidance and robust decision-making – and reduce the cost where it matters least: ritualistic assessment with little incremental or learner relevant information, trying to optimize individual data points in all aspects of reliability, validity and educational consequences. In our view, any assessment is an optimization problem [30]. It is time to include economic arguments into the optimization puzzle.
